# Supplementary material for: Rewiring of Cancer Cell Metabolism by Mitochondrial VDAC1 Depletion Results in Time-Dependent Tumor Reprogramming: Glioblastoma as a Proof of Concept
Source: Cells. 2019 Oct 28;8(11):1330. doi: 10.3390/cells8111330 (PMC6912264; doi:10.3390/cells8111330)
Supplement: Supplementary file 1 [file cells-08-01330-s001.pdf]

Supplementary Data

Materials and Methods

Materials

The cell transfection agents JetPRIME and JetPEI were obtained from PolyPlus Transfection (Illkirch, France), while non-modified and 2′-O-methyl-modified hVDAC1-siRNAs were obtained from Genepharma (Suzhou, China). Bovine serum albumin (BSA), Triton X-100, Tween-20, hematoxylin, eosin and 4′,6-diamidino-2-phenylindole (DAPI) were obtained from Sigma (St. Louis, MO). Paraformaldehyde was purchased from Emsdiasum (Hatfield, PA). Dulbecco's modified Eagle's medium (DMEM), and Roswell Park Memorial Institute (RPMI) 1640 growth medium were obtained from Gibco (Grand Island, NY). Normal goat serum (NGS), fetal calf serum (FCS) and the supplements L-glutamine and penicillin-streptomycin were obtained from Biological Industries (Beit Haemek, Israel). Primary antibodies, their sources and the dilutions used are detailed in Supplementary Table S1. Horseradish peroxidase (HRP)-conjugated anti-mouse, anti-rabbit and anti-goat antibodies were from KPL (Gaithersburg, MD). 3,3-diaminobenzidine (DAB) was obtained from ImmPact-DAB (Burlingame, CA).

Immunoblotting

Tumor tissues were solubilized in a lysis buffer (50 mM Tris-HCl, pH 7.5, 150 mM NaCl, 1 mM EDTA, 1.5 mM $MgCl_2$, 10% glycerol, 1% Triton X-100, a protease inhibitor cocktail (Calbiochem)), followed by sonication and centrifugation (10 min, 600 g). The protein concentration of each lysate was determined using a Lowry assay. Samples were stored at -80^{0}C until analysis by immunoblotting.

For immunostaining, membranes containing electro-transferred proteins following SDS-PAGE were blocked with 5% non-fat dry milk and 0.1% Tween-20 in TBS, incubated with the primary antibodies (sources and dilutions as detailed in Supplementary Table S1) and then with HRP-conjugated anti-mouse or anti-rabbit (1:10,000) or anti-goat (1:20,000) IgG. Blots were developed using enhanced chemiluminescence (Biological Industries). Band intensities were analyzed by densitometry using FUSION-FX (Vilber Lourmat, France) software and values were normalized to the intensities of the appropriate ⊚-actin signal that served as a loading control.

Immunohistochemistry (IHC) and immunofluorescence analysis of tumor tissue sections

Immunohistochemical and immunofluorescence staining were performed on 5 μm-thick formalin-fixed and paraffin-embedded tumor tissue sections. The sections were deparaffinized by placing the slides at 60°C for 1 h and using xylene. Thereafter, the tissue sections were rehydrated with a graded ethanol series (100-50%). Antigen retrieval for some proteins (i.e., VDAC1, Glut1, ATP5a, caspase 3, , Cyto *c*, TSPO, AIF, TUBB3, GFAP was performed in 0.01 M citrate buffer (pH 6.0) for 30 minutes at 95-98°C. After washing the sections in PBS containing 0.1% Triton-X100 (pH 7.4), non-specific antibody binding was reduced by incubating the sections in 10% NGS for 2 h. After decanting excess serum, the sections were incubated overnight at 4°C with primary antibodies (sources and dilutions used are detailed in Table S1). Sections were washed with phosphate-buffered saline with 0.1% Tween-20 (PBST). For IHC, endogenous peroxidase activity was blocked by incubating the sections in 3% H_2O_2 for 10 min. After washing thoroughly with PBST, the sections were incubated for 2 h with anti-mouse or anti-rabbit (1:500) secondary antibodies conjugated to HRP. Sections were washed in PBST and peroxidase activity was visualized by incubating with 3,3-diaminobenzidine (DAB) (ImmPact-DAB, Burlingame, CA). After rinsing in water, the sections were counterstained with hematoxylin, and mounted with mounting medium. Finally, the sections were observed under a microscope (Leica DM2500) and images were collected at 20× magnification with the same light intensity and exposure time. Non-specific control experiments were carried out using the same protocols but omitting incubation with primary antibodies. For immunofluorescence, alexa flour 488-conjugated anti-rabbit (1:500) or Alexa flour 555-conjugated anti-mouse (1:500) secondary antibodies were used. The cells were then stained with DAPI (0.07 μg/ml) and viewed with an Olympus IX81 confocal microscope.

Quantitative analysis: Quantification of the immunostained images generated was carried out using HistoQuant software (Quant Center 2.0 software, 3DHISTECH Ltd, Hungary).

RNA preparation and qRT-PCR

Total RNA was isolated from si-NT-treated tumors (TTs) and si-hVDAC1-TTs (4 mice each) using an RNeasy mini kit (Qiagen) according to the manufacturer's instructions. Total RNA quality was analyzed using the Agilent

RNA 6000 nano kit. The RNA integrity values obtained for total RNA extracted from si-NT-TTs and si-hVDAC1-TTs were 8-10 and 7-8.5, respectively. Total RNA was isolated as above. Complementary DNA was synthesized from 1 μg total RNA using a Verso cDNA synthesis kit (Thermo Scientific). qRT-PCR was performed using specific primers (KiCqStart Primers; Sigma Aldrich) in triplicate, using Power SYBER green master mix (Applied Biosystems, Foster City, CA). The levels of the target genes were normalized relative to ⊚-actin mRNA levels. Samples were amplified by a 7300 Real Time PCR System (Applied Biosystems) for 40 cycles using the following PCR parameters: 95°C for 15 seconds, 60°C for 1 minute, and 72°C for 1 minute. The copy numbers for each sample were calculated by the CT-based calibrated standard curve method. The mean fold changes (± SEM) of the three replicates were calculated. Genes examined and primers used are listed in Supplementary Table S2.

Liquid chromatography–high-resolution mass spectrometry (LC-HR-MS/MS) and proteomics analysis

To extract proteins for LC-HR MS/MS analysis, tumor tissues were solubilized in lysis buffer (100 mM Tris-HCl, pH 8.0, 5 mM DTT 4% SDS and a protease inhibitor cocktail (Calbiochem)), followed by homogenization, incubation for 3 min at 95°C and centrifugation (10 min, 15,000 g). The protein concentration of each lysate was determined using a Lowry assay. Samples were stored at -80 ^{0}C until LC-HR-MS/MS analysis.

Mass spectrometry (MS) proteomics profiling and initial processing of the results were carried out at the de Botton Institute for Protein Profiling, G-INCPM, Weizmann Institute of Science. Samples were subjected to in-solution tryptic digestion as follows. Proteins were first reduced by incubation with 5 mM DTT for 30 min at 60°C, followed by alkylation with 10 mM iodoacetamide in the dark for 30 min at 21°C. Proteins were then subjected to digestion with trypsin (Promega, Madison, WI) at a 1:50 trypsin:protein ratio for 16 h at 37°C. Following digestion, detergents were cleared from the samples using commercial detergent removal columns (Pierce, Rockford, IL), and the samples were desalted using solid-phase extraction columns (Oasis HLB, Waters, Milford, MA). Digestions were stopped by addition of trifluroacetic acid (1%). The samples were stored at -80˚C until LC-HR MS/MS analysis.

For LC-HR MS/MS, MS grade solvents were used for all chromatographic steps. Each sample was separated using split-less nano-ultra performance liquid chromatography columns (10 kpsi nanoAcquity; Waters). The mobile phase was (A) H_2O and 0.1% formic acid, and (B) acetonitrile and 0.1% formic acid. Desalting of the samples was performed online using a reverse-phase C18 trapping column (180 μm internal diameter, 20 mm length, 5 μm particle size; Waters). The peptides were then separated using a T3 HSS nano-column (75 μm internal diameter, 250 mm length, 1.8 μm particle size; Waters) at 0.3 μL/min. Peptides were eluted from the column into the mass spectrometer using the following gradient: 4% to 35% (B) for 150 min, 35% to 90% (B) for 5 min, maintained at 90% for 5 min and then back to initial conditions. The nano-UPLC (Ultra High-Performance Liquid Chromatography) was coupled online through a nano-ESI (Electrospray Ionization) emitter (10 μm tip; New Objective, Woburn, MA) to a quadrupole Orbitrap mass spectrometer (Q Executive, Thermo Scientific) using a FlexIon nanospray apparatus (Proxeon). Data were acquired in the DDA (data-dependent acquisition) mode, using the Top12 method (1). Raw data were imported into Expressionist software (Genedata) (2,3). The software was used for retention time alignment and peak detection of precursor peptide intensities. A master peak list was generated from all MS/MS events and sent for database searching using Mascot v2.4 (Matrix Sciences). Data were searched against a database containing forward and reverse human and mouse protein sequences from UniprotKB/SwissProt, and 125 common laboratory contaminants, totaling 20,304 entries. Fixed modification was set to carbamidomethylation of cysteines, while variable modification was set to oxidation of methionines. Search results were then imported back to Expressionist for annotation of detected peaks. Identifications were filtered such that the global false discovery rate was a maximum of 1%. Protein abundance was calculated based on the three most abundant peptides (4).

Table S1. Antibodies used in this study

Antibodies against the indicated protein, their catalogue number, source, and the dilutions used in IHC, immunoblot and immunofluorescence experiments are presented.

Antibody	Source and Cat. No.	Dilution		
		IHC	WB	IF
Rabbit polyclonal anti-AIF	R&D Systems, Inc. MN, USA, AF1457	1:300	-	-
Rabbit monoclonal anti-ALDH1	Abcam, Cambridge, UK, ab52492	1:200	1:2000	-
Mouse monoclonal anti-ATP5a	Abcam, Cambridge, UK, ab14748	1:300	1:1000	-
Mouse monoclonal anti-β-actin	Millipore, Billerica, MA, MAB1501	-	1:10000	-
Rabbit polyclonal anti-Caspase3	Cell Signaling Technology, Danvers, MA, #9662	1:300	-	-
Mouse monoclonal anti-CD133	Miltenyi Biotec GmbH, AC133	-	1:1500	-
Rabbit polyclonal anti-CD44	Abcam, Cambridge, UK ab157107	1:100	1:3000	-
Rabbit polyclonal anti-citrate synthase	Abcam, Cambridge, UK ab96600	1:200	1:4000	-
Rabbit monoclonal cytochrome c oxidase subunit VIc	Abcam, Cambridge, UK, ab150422	1:200	1:2000	-
Mouse monoclonal anti-GAD-67	Abcam, Cambridge, UK, ab26116	-	-	1:1500
Mouse monoclonal anti-GAPDH	Abcam, Cambridge, UK, ab9484	1: 200	1:1000	-
Mouse monoclonal anti-GFAP	Santa Cruz Biotechnology, Inc. Dallas, TX, sc-33673	-	1:2000	1:200
Rabbit monoclonal anti-Glut1	Abcam, Cambridge, UK ab40084	1: 200	1: 1500	-
Mouse monoclonal anti-HK-I	Abcam, Cambridge, UK ab105213	1:500	1:2000	-
Rabbit polyclonal anti-KLF4	IMGENX Littleton, USA, IMG-6081-A	1:200	1:1000	-
Goat polyclonal anti-LDH-A	Santa Cruz Biotechnology, Inc. Dallas, TX, sc-27230	-	1:1500	-
Rabbit polyclonal anti-Nestin	Millipore, Billerica, MA, MAB353	1:400	1:1000	-
Rabbit monoclonal anti-PBR (TSPO)	Abcam, Cambridge, UK ab109497	1:200	-	1:300
Goat polyclonal anti-SOX2	Santa Cruz Biotechnology, Inc. Dallas, TX, sc-17320	1:200	1:1500	-
Mouse monoclonal anti-beta III tubulin	Abcam, Cambridge, UK ab7751	-	1:2000	1:200
Rabbit monoclonal anti-VDAC1	Abcam, Cambridge, UK , ab154856	1:500	1:5000	-

Table S2. Real-Time PCR primers used in this study

The genes examined, and the forward and reverse sequences of the primers used are indicated.

Gene	Primer sequences
ß-Actin	Forward 5′-ACTCTTCCAGCCTTCCTTCC-3′<br>Reverse 5′- TGTTGGCGTACAGGTCTTTG-3′
ALDH1L1	Forward 5′-CCAAAGTCCTGGAGGTTGAA-3′<br>Reverse 5′-TAACTCCAGGCCATCACACA-3′
ATP5a syn	Forward 5′-TCAGTCTACGCCGCACTTAC-3′<br>Reverse 5′- GACATCTCAGCAGTCCCACA-3′
CD133	Forward 5′-TGGGCTTGTCATAACAGGAT-3′<br>Reverse 5′-TTGCGGTAAAACTGGCTAAG-3′
CS	Forward 5′- AGGAACAGGTATCTTGGCTCT-3′<br>Reverse 5′- GGGGTGTAGATTGGTGGGAA-3′
GFAP	Forward 5′- AAGCTCCAGGATGAAACCAAC-3′<br>Reverse 5′- AGCGACTCAATCTTCCTCTCC-3′
GAPDH	Forward 5′- TGGAAGGACTCATGACCACA-3′<br>Reverse 5′- ATGATGTTCTGGAGAGCCCC-3′
GLUT1	Forward 5′- GGCCATCTTTTCTGTTGGGG-3′<br>Reverse 5′- TCAGCATTGAATTCCGCCG-3′
HK-I	Forward 5′-GTCTCAGTCCAGCACGTTTG-3′<br>Reverse 5′- GAAACGCCGGGAATACTGTG-3′
LDH-A	Forward 5′-GCARabbitGGTGGTTGAGAGTGCTT-3′<br>Reverse 5′- GCACCCGCCTAAGATTCTTC-3′
MAP2	Forward 5′-TCCAAAATCGGATCAACAGAC-3′<br>Reverse 5′- AGAGCCACATTTGGATGTCAC-3′
Nanog	Forward 5′- TGGGATTTACAGGCGTGAGCCAC-3′<br>Reverse 5′- AAGCAAAGCCTCCCAATCCCAAAC-3′
Nestin	Forward 5′- GAAACAGCCATAGAGGGCAAA-3′<br>Reverse 5′- TGGTTTTCCAGAGTCTTCAGTGA-3′
Oct3/4	Forward 5′ GGGCTCTCCCATGCATTCAAAC-3′<br>Reverse 5′-CACCTTCCCTCCAACCAGTTGC -3′
SOX2	Forward 5′- CCATGCAGGTTGACACCGTTG -3′<br>Reverse 5′-TCGGCAGACTGATTCAAATAA-3′
TUBB3	Forward 5′-CTCAGGGGCCTTTGGACATC-3′<br>Reverse 5′-CAGGCAGTCGCAGTTTTCAC-3′
VDAC1	Forward 5′-AGACTGCAAAATCCCGAGTG-3′<br>Reverse 5′-CCAAACTCTGTCCCGTCATT-3′

Abbreviations used in the following Tables

AML, acute myeloid leukemia; CRC, colorectal cancer; CRCA, colorectal carcinoma; CSCs, cancer stem cells, ECM, extracellular matrix; EMT, epithelial-mesenchymal transition, ER: endoplasmic reticulum; ESCC, esophageal squamous cell carcinoma ; GBM, glioblastoma multiforme; GPCR, G protein-coupled receptor; HCC, hepatocelular carcinoma; HNSCC, head and neck squamous cell carcinoma; LUAD: lung adenocarcinoma; NPC, nuclear pore complex; NSCLC, non-small cell lung cancer; OC, ovarian carcinoma; OPN, osteopontin; PCNA: proliferating cell nuclear antigen; RCC, renal cell carcinoma; SCC, squamous cell carcinoma; TCA, tricarboxylic acid cycle; VEGF, vascular endothelial growth factor.

Table S3. Alterations in the expression of metabolism-related human proteins in GBM xenografts treated for short and long periods with si-hVDAC1

LC-HR MS/MS analysis was performed as described in the Materials and Methods section. Differentially expressed proteins (p-value <0.05, FC ǀ1.5ǀ) were filtered and those proteins differentially expressed in tumors treated with si-NT- or si-hVDAC1 for short or long periods are presented. For each protein, the name, fold change (FC) and p-value, as well as its function, subcellular localization and relevance to cancer, are indicated.

No.	**Protein name** (UniProtKB)	**Proposed function (cell localization)**	**Short-term treatment si-hVDAC1/ si-NT FC (P value)**	**Long-term treatment si-hVDAC1/ si-NT FC (P value)**	**Relation to cancer**
1.	**ABHD10 - α/β Hydrolase domain-containing 10** (Q9NUJ1)	Catalyzes the deglucuronidation of mycophenolic acid acyl-glucuronide, a metabolite of the immunosuppressant drug mycophenolate (mitochondria)	-1.66 (0.529368)	-42.45 (0.020634)	Exceptional high expression of α/β hydrolase domain containing 6 (ABHD6) in Ewing family tumors (EFT) but not in other sarcomas (5)
2.	**ADSS – Adenylosuccinate synthetase isozyme 2** (P30520)	Catalyzes the first of two steps in the synthesis of AMP from IMP	2.99 (0.057386)	-14.78 (0.026987)	Up-regulation is associated with poor prognosis in GBM patients (6)
3.	**ALDH2 - Aldehyde dehydrogenase 2** (P05091)	Synthesizes acetate from ethanol (mitochondria)	1.13 (0.849539)	9.24 (0.023517)	Involved in stemness and resistance to microtubule inhibitors in lung, head and neck cancer cells (7)
4.	**CS - Citrate synthase** (O75390)	Catalyzes the first step in TCA cycle, the synthesis of isocitrate from oxaloacetate and acetyl CoA (mitochondria)	-1.17 (0.452195)	-2.4 (0.0113717)	In cervical carcinoma cancer cells, loss of CS switches the cell from mitochondrial respiration to glycolysis and induces EMT phenotype (8)
5.	**FDXR - Ferredoxin reductase** (P22570)	Transfers electrons from NADPH to cytochrome P450. Involved in metabolic processes, such as the synthesis of bile acid, iron–sulfur clusters, vitamin D, and steroid hormones (mitochondria)	5.53 (0.0186192)	5.73 (0.0371625)	FDXR is required for p53-dependent tumor suppression via iron homeostasis (9)
6.	**GPD1L - Glycerol-3-phosphate dehydrogenase 1-like protein** (Q8N335)	Catalyzes the conversion of sn-glycerol 3-phosphate to glycerone phosphate (cytoplasm)	-2.14 (0.110365)	-10.25 (0.0034338)	Significantly down-regulated in head and neck squamous cell carcinoma (10)
7.	**NT5E/CD73 - 5'-nucleotidase** (P21589)	Hydrolyzes extracellular 5′-adenosine monophosphate to membrane-permeable adenosine (plasma membrane)	-1.61 (0.163684)	-5.53 (0.0034471)	Marker for chemo-resistance in GBM (11). Promotes HCC growth and metastasis mediated by EGFR (12). High levels of CD73 expression are associated with early) recurrence in HNSCC patients (13).
8.	**HCR-NTPase - Human cancer-related nucleoside-triphosphatase** (**NTPase**) (Q9BSD7)	Non-specific nucleoside triphosphatase	3.07 (0.0468058)	1.23 (0.724411)	Over-expression in neuroblastoma cell line led to increased cytotoxicity (14)

9.	**OXCT1 - 3-oxoacid CoA-transferase 1** (P55809)	Involved in ketone body catabolism. Transfers CoA from succinate to acetoacetate (mitochondria)	-1.08 (0.748254)	3.9 (0.0036903)	Up-regulated in HCC, NSCLC and prostate cancer (15)
10.	**PC - Pyruvate carboxylase** (P11498)	Catalyzes the ATP-dependent carboxylation of pyruvate to form oxaloacetate (mitochondria)	1.06 (0.869796)	-3.49 (0.0178811)	Up-regulated in breast cancer and essential for growth and invasion of MDA-MB-231 cells (16)
11.	**PYCR3/PYCRL – Pyrroline-5 carboxylate reductase 3** (Q53H96)	Participates in production of proline from ornithine (cytosol)	1.03 (0.923686)	-2.92 (0.016966)	Promotes proliferation and inhibits apoptosis in non-small cell lung cancer (17)
12.	**SDHA - Succinate dehydrogenase** (P31040)	A subunit of succinate SDH, involved in complex II of the mitochondrial electron transport chain (mitochondria)	1.23 (0.619825)	-3.59 (0.0398968)	Acts as tumor suppressor. Deficiency is associated with hereditary paraganglioma and pheochromocytoma (18)
13.	**TIGAR -TP53-induced glycolysis and apoptosis regulator** (Q9NQ88)	Negative regulator of glycolysis, hydrolyzes fructose-2,6-bisphosphate and fructose-1,6-bisphosphate	1.6 (0.670544)	37.63 (0.0243289)	Over-expressed in colon and breast cancers and glioblastoma (19)

Table S4. **Alterations in the expression of transport- and trafficking-related human proteins in GBM xenografts treated for short and long periods with si-hVDAC1**

LC-HR MS/MS analysis was performed as described in the Materials and Methods section. Differentially expressed proteins (p-value <0.05, FC |1.5|) were filtered and those proteins differentially expressed in tumors treated with si-NT- or si-hVDAC1 for short or long periods are presented. For each protein, the name, fold change (FC) and p-value, as well as its function, subcellular localization and relevance to cancer, are indicated.

No.	Protein name (UniProtKB)	Proposed function (cell localization)	Short-term treatment si-hVDAC1/ si-NT FC (P value)	Long-term treatment si-hVDAC1/ si-NT FC (P value)	Relation to cancer
**Transport**					
1	**ATP2B1 – Plasma membrane calcium-transporting ATPase 1 / PMCA1** (P20020)	Mediates calcium export through plasma membrane (plasma membrane)	-1.21 (0.812652)	-52.04 (0.0171061)	Down-regulated in oral cancer (20)
2	**ARL6IP1 - ADP-ribosylation factor-like protein 6-interacting protein 1** (Q15041)	Improves glutamate transport (ER)	1.19 (0.77814)	-10.66 (0.0098373)	Down-regulation suppressed tumor cell proliferation and invasion in cervical cancer (21)
3	**GLUT1 - Solute carrier family 2, facilitated glucose transporter member 1** (P11166)	Responsible for constitutive or basal glucose uptake (plasma membrane)	-2.48 (0.34905)	-19.37 (0.0355712)	Over-expressed in many cancers, including hepatic, pancreatic, breast, esophageal, brain, renal, lung, cutaneous, colorectal, endometrial, ovarian and cervical cancers (22). High expression of GLUT1 is associated with poor survival in GBM, CRCA, LAUD, SCC and OC (23)
4	**LETM1 - Leucine zipper–EF-hand-containing transmembrane protein 1** (O95202)	Mediates proton-dependent K^+ and Na^+ efflux from mitochondrion, thereby indirectly modulating Ca^{2+} fluxes (mitochondria)	1.24 (0.52466)	4.24 (0.0103477)	Marker of CSCs and a predictor of poor prognosis in esophageal squamous cell carcinoma (24)
5	**MAGT1 - Magnesium transporter protein 1** (Q9H0U3)	Magnesium ion transporter (plasma membrane)	-1.3 (0.33102)	-2.16 (0.0439223)	Chemoresistance to doxorubicin is characterized by its up-regulation in colon carcinoma cells (25)
6	**SLC39A14 - Zinc transporter ZIP14** (Q15043)	Metal ion transporter. Facilitates zinc uptake (plasma membrane)	-1.16 (0.735453)	-4.89 (0.0207008)	Down-regulated in hepatoma cells, leading to depletion of zinc in hepatoma cells in HCC (26)
**Trafficking**					
7	**ARFGEF2 - Brefeldin A-inhibited guanine nucleotide exchange protein 2 (BIG2)** (Q9Y6D5)	Trafficking endosomes between the trans-Golgi network and endosomes (endosome, Golgi)	1.32 (0.571388)	-47.74 (0.0010678)	None reported
8	**EXOC1 - Exocyst complex component 1 (SEC3)** (Q9NV70)	Involved in vesicle tethering during exocytosis (cytosol)	-1.65 (0.382779)	-21.08 (0.0033224)	None reported

9	**GOLGA5 / GOLGIN84 - Golgin subfamily A member 5 (**Q8TBA6)	Involved in maintaining Golgi structure and in intra-Golgi retrograde transport (Golgi)	-2.02 (0.103283)	-16.47 (0.0083353)	Correlated with poor survival in metastatic colorectal adenocarcinoma (27)
10	**GOPC / PIST - Golgi-associated PDZ and coiled-coil motif-containing protein** (Q9HD26)	Controls the trafficking of integral membrane proteins from the trans-Golgi network to the cell surface (Golgi, plasma membrane)	-1.34 (0.52616)	3.85 (0.0440635)	Low expression of the GOPC is a poor prognostic marker in colorectal cancer. Mutated in GBM (28)
11	**NUP205 - Nucleoporin 205** (Q92621)	A subunit of the NUP93 complex, the second largest NPC structural unit (nucleus)	-1.40 (0.0624531)	-2.05 (0.0070202)	Proposed as retinoblastoma driver candidates (29)
12	**NUP88 - Nucleoporin 88 (**Q99567)	A nucleoporin located on the cytoplasmic face of the NPC. Participates in nuclear export (nucleus)	-2.10 (0.407481)	-18.6 (0.0283846)	Over-expressed and accumulates in the cytoplasm in ovarian, breast and colorectal cancers (30,31)
13	**NUP93 - Nucleoporin 93** (Q8N1F7)	An adaptor nucleoporin that associates with other subunits of the Nup93 subcomplex, involved in assembly and permeability maintenance of the NPC (nucleus)	-1.04 (0.904658)	-10.83 (0.0011731)	Mutation in *NUP93* increased tumor cell migration, and metastasis in breast cancer (32)
14	**NXF1 - Nuclear RNA export factor 1** (Q9UBU9)	Involved in the nuclear export of mRNA species (nucleus)	-2.46 (0.0436826)	-5.59 (0.0073036)	Predicted to be a tumor suppressor protein (33)
15	**PSD3 - Pleckstrin and Sec domain-containing 3 / EFA6R -** (Q9NYI0)	Guanine nucleotide exchange factor for the small GTPase ARF6 that regulates membrane trafficking (plasma membrane)	-1.17 (0.74844)	-10.39 (0.0062725)	Down-regulated in high grade ovarian carcinomas and primary glioblastomas, relative to lower grade ovarian and glioma tumors (34)
16	**RAB12 - Ras-related protein Rab-12** (Q6IQ22)	Trafficking of vesicles within the cell (endosome, Golgi, lysosome)	-1.63 (0.335603)	-9.29 (0.0066509)	None reported Remove this Ref
17	**α-SNAP- Alpha-soluble NSF attachment protein** (P54920)	Transport of vesicles between the ER and the Golgi apparatus (cytosol)	-1.24 (0.466453)	-2.75 (0.025271)	Changes in expression level contribute to aggressive neuroendocrine tumors (35)
18	**STEAP3 - Metalloreductase STEAP3** (Q658P3)	Endosomal ferrireductase required for reduction of Fe^{3+} to Fe^{2+} (endosome)	-1.36 (0.535821)	-4.3 (0.0446126)	Promotes growth and invasion in GBM (36)
19	**TOM70 - Import receptor subunit TOM70** (O94826)	Import of mitochondrial precursor proteins (mitochondria)	-1.51 (0.102457)	-2.7 (0.0092339)	Overexpressed in mitochondria of breast cancer cells (37)
20	**TRAPPC8 - Trafficking protein particle complex subunit 8** (Q9Y2L5)	Trafficking from ER to Golgi apparatus at a very early stage (Golgi)	1.24 (0.333497)	-2.16 (0.0222307)	None reported
21	**TSG101 - Tumor susceptibility gene 101 protein** (Q99816)	Component of the ESCRT-I complex, a regulator of vesicular trafficking process (cytoskeleton, endosome, nucleus)	-1.08 (0.649469)	-1.96 (0.0158835)	Up-regulated in papillary thyroid carcinomas (38) and in a subset of invasive human breast cancers (39)

Table S5. Alterations in the expression of signaling pathways, development- and differentiation-related human proteins in GBM xenografts treated for short and long periods with si-hVDAC1

LC-HR MS/MS analysis was performed as described in the Materials and Methods section. Differentially expressed proteins (p-value <0.05, FC |1.5|) were filtered and those proteins differentially expressed in tumors treated with si-NT- or si-hVDAC1 for short or long periods are presented. For each protein, the name, fold change (FC) and p-value, as well as its function, subcellular localization and relevance to cancer, are indicated.

No.	**Protein name** (UniProtKB)	**Proposed function (cell localization)**	**Short-term treatment si-hVDAC1/ si-NT FC (P value)**	**Long-term treatment si-hVDAC1/ si-NT FC (P value)**	**Relation to cancer**
1	**CPNE3 - Copine-3** (O75131)	A calcium-dependent membrane-binding protein that participates in several biological functions at the interface of the cell membrane and the cytoplasm (plasma membrane, nucleus)	-1.84 (0.1312)	-3.42 (0.0284455)	Promotes tumor cell migration upon interaction with ErbB2 in breast cancer cells (40). Up-regulated in prostate and ovarian tumors (40)
2	**ICAM1 – Inter-cellular adhesion molecule 1** (P05362)	Facilitates leukocyte endothelial transmigration with a pro-inflammatory effect (plasma membrane)	-1.17 (0.772779)	-16.06 (0.005036)	Over-expressed in breast cancer (41), mediates tumor metastasis in pancreatic ductal adenocarcinoma (42)
3	**LANCL2 - Lanthionine Synthetase C-like Receptor 2** (Q9NS86)	Negative regulator of inflammatory pathways. Promotes immuno-regulatory responses characterized by IL-10 production (plasma membrane, cytosol, nucleus)	-1.94 (0.394598)	-10.3 (0.0393035)	Increases cellular sensitivity to adriamycin in glioblastomas (43)
4	**MFN2 - Mitofusin-2** (O95140)	Mitochondrial outer membrane GTPase that mediates mitochondrial clustering and fusion (mitochondria)	6.72 (0.026583)	-1.64 (0.559993)	Low MFN2 expression in many types of cancer is associated with poor prognosis (44)
5	**PGAM5 -Serine/ threonine protein phosphatase** (Q96HS1)	Dephosphorylates and activates ASK1 kinase (mitochondria)	-1.03 (0.882268)	-4.87 (0.0008375)	Up-regulated in HCC and associated with poor prognosis. Enhances chemo-resistance (45)
6	**PLXNB2 - Plexin-B2** (O15031)	Transmembrane receptor for semaphorins that participates in axon guidance and cell migration during development (plasma membrane)	-1.18 (0.750354)	-5.38 (0.0343065)	Promotes invasive growth and vascularization of malignant gliomas (46)
7	**RHEB - GTP-binding protein Rheb** (Q15382)	Activates cell growth and proliferation by activating mTOR (nucleus, cytoplasm)	-1.42 (0.356984)	-2.96 (0.0444695)	Prolongs survival of colon cancer cells (47)
8	**ROCK1 - Rho-associated protein kinase 1** (Q13464)	A serine-threonine kinase, a downstream effector of Rho GTPases, acts as a suppressor of inflammatory cell migration by regulating PTEN phosphorylation and stability (plasma membrane, nucleus)	4.65 (0.0881424)	-14.12 (0.0208807)	Involved in breast cancer progression and invasion (48)
9	**RRAD - Ras-related associated with diabetes** (P55042)	p53-target. Represses glycolysis through inhibition of GLUT1 translocation to the plasma membrane (plasma membrane	2.24 (0.195629)	12.74 (0.0077209)	Frequently down-regulated in lung cancer, associated with tumor progression and poor prognosis (49), hepatoma cell invasion and metastasis (50)

10	**STAT1 – Signal transducer and activator of transcription 1-alpha/beta** (P42224)	Regulates gene expressions and promotes cell death, inhibits cell growth, immune cell stimulation and cell differentiation (cytosol, nucleus)	-1.13 (0.4881)	-1.74 (0.0357768)	Inhibits tumorigenesis of mammary adenocarcinoma and ovarian teratomas (51,52)
11	**STAT6 - Signal transducer and activator of transcription 6** (P42226)	Transcription activation. Involved in IL4- and IL13-mediated signaling (cytosol, nucleus).	2.12 (0.222314)	11.5 (0.0112681)	Involved in EMT in colorectal cancer (53)
12	**SUSD2 - Sushi domain-containing protein 2** (Q9UGT4)	Promotes cell adhesion and migration (plasma membrane)	3.71 (0.199269)	154.29 (0.0039901)	Suppresses metastasis of high-grade serous ovarian carcinoma (54) and promotes metastasis and cisplatin resistance in serous ovarian cancer (55). Promotes proliferation and clonogenicity of RCC (56)
**Development and Differentiation**					
13	**APMAP-Adipocyte plasma membrane-associated protein** (Q9HDC9)	Functions in adipogenesis. Up-regulated during adipogenic differentiation (plasma membrane)	-1.62 (0.0504605)	-2.32 (0.0131208)	None reported
14	**MYLK - Myosin light chain kinase, smooth muscle** (Q15746)	Ca^{2+}/calmodulin-dependent myosin light chain kinase that phosphorylates the regulatory light chain to initiate contraction in smooth muscle (cytoskeleton)	2.20 (0.0443153)	-1.11 (0.784895)	Promotes EMT and migration of HCC cells (57)
15	**NRP1 - Neuropilin-1** (O14786)	Membrane-bound isoform 1 is a receptor for class 3 semaphorins in neurons, and for VEGF in vascular endothelial cells. Involved in axonal guidance (plasma membrane)	-1.33 (0.612255)	-5.66 (0.0367593)	Involved in the development and progression of melanoma, astrocytoma, glioma and neuroblastoma. Facilitates proliferation and inhibits apoptosis of glioma cells (58,59)
16	**PRDX4-Peroxiredoxin-4** (Q13162)	Thiol-specific peroxidase that catalyzes reduction of hydrogen peroxide and organic hydroperoxides to water and alcohols. Regulates neurogenesis (ER, secreted)	-1.12 (0.738317)	-4.19 (0.0116516)	Promotes metastasis of breast and prostate cancer (60)

Table S6. Alterations in the expression of ECM-related mouse proteins in GBM xenografts treated for short and long periods with si-hVDAC1

LC-HR MS/MS analysis was performed as described in the Materials and Methods section. Differentially expressed proteins (p-value <0.05, FC |1.5|) were filtered and those proteins differentially expressed in tumors treated with si-NT- or si-hVDAC1 for short or long periods are presented. For each protein, the name, fold change (FC) and p-value, as well as its function, subcellular localization and relevance to cancer, are indicated.

No.	**Protein name** (UniProtKB)	**Proposed function (cell localization)**	**Short-term treatment si-hVDAC1/ si-NT FC (P value)**	**Long-term treatment si-hVDAC1/ si-NT FC (P value)**	**Relation to cancer**
1	**CD47 Integrin-associated protein** (Q61735)	Involved in modulation of integrins and cell adhesion to extracellular matrix (plasma membrane)	-2.06 (0.0493541)	-3.33 (0.0157124)	Enables cancer cell escape from immune surveillance. A target for GBM immunotherapy (61)
2	**EMB Embigin** (P21995)	Regulates neural cell adhesion and cell-ECM interactions during development. Involved in prostate and mammary gland development (plasma membrane)	-1.26 (0.676742)	-6.38 (0.0298455)	Promotes prostate cancer progression (62) Over-expressed in pancreatic ductal adenocarcinoma and regulates cell motility (63)
3	**MMP8 Neutrophil collagenase** (O70138)	Cleaves interstitial collagens (types I, II, and III) in the triple helical domain (secreted protein)	-2.70 (0.0337055)	-11.64 (0.00148073)	Decreases skin tumorigenesis and may reduce breast cancer metastasis (64)
4	**MMP9 Matrix metalloproteinase-9** (P41245)	Cleaves type IV and type V collagen (secreted protein)	-1.66 (0.217068)	-18.48 (0.000647019)	Exerts tumor-promoting effects at early stage of skin carcinogenesis, and anti-cancer effects at later stages (64)
5	**MYH10 Myosin-10** (Q61879)	Plays important roles in cytoskeleton reorganization, focal contact formation and lamellipodial extensions, controls stability of mitochondrial DNA	4.14 (0.283047)	79.84 (0.0250541)	Enhanced expression in platelets is a marker of acute myeloid leukemia or chronic myelomonocytic leukemia (65)
6	**HMGB2 High mobility group protein B2** (P30681)	Promotes pro-inflammatory conditions (secreted protein)	-1.21 (0.627301)	-6.99 (0.0051323)	Associated with poor prognosis of pancreatic cancer. Promotes cell proliferation and survival (66)

Table S7. Alterations in the expression of ECM related human proteins in GBM xenografts treated for short and long periods with si-hVDAC1

LC-HR MS/MS analysis was performed as described in the Materials and Methods section. Differentially expressed proteins (p-value <0.05, FC |1.5|) were filtered and those proteins differentially expressed in tumors treated with si-NT- or si-hVDAC1 for short or long periods are presented. For each protein, the name, fold change (FC) and p-value, as well as its function, subcellular localization and relevance to cancer, are indicated

No.	**Protein name** (UniProtKB)	**Proposed function (cell localization)**	**Short-term treatment si-hVDAC1/ si-NT FC (P value)**	**Long-term treatment si-hVDAC1/ si-NT FC (P value)**	**Relation to cancer**
1	**CD44 - CD44 antigen** (P16070)	Receptor for hyaluronic acid (HA), matrix metalloproteases and collagens. Mediates cell-cell and cell-matrix interactions and cell adhesion (plasma membrane)	-1.19 (0.501414)	-2.42 (0.0228934)	Induces stemness and EMT in gliomas and breast cancer (67,68). CD44 is a marker of metastasis in colon and gastric cancer (68)
2	**GPC1 – Glypican-1** (P35052)	Major constituent of the ECM. A proteoglycan that influences cell proliferation, differentiation and gene expression (endosome, Golgi, nucleus, plasma membrane)	-1.31 (0.39608)	-4.21 (0.00765675)	Up-regulated in human cancers, such as glioma, pancreatic and breast cancers (69). Marker of colorectal cancer (70)
3	**ITGA3 - Integrin alpha-3** (P26006)	An integrin subunit that with a β1 subunit forms an integrin receptor for fibronectin, laminin, collagen, epiligrin, thrombospondin and CSPG4 (plasma membrane)	-1.24 (0.496356)	-2.69 (0.0368846)	Promotes tumor cell migration and invasion in HNSCC (71)
4	**ITGA5 - Integrin alpha-5** (P08648)	An integrin alpha-5/beta-1 that is a receptor for fibronectin and fibrinogen (plasma membrane)	-1.11 (0.735803)	-2.88 (0.0293)	Promotes migration, invasion, and proliferation in GBM (72). High expression is associated with poor overall survival in NSCLC (73)
5	**LGALS1- Galectin-1 / Gal-1** (P09382)	Binds to ECM glycoconjugates like laminin and fibronectin. Regulates cell proliferation, migration, apoptosis and angiogenesis. Involved in cell cycle regulation and resolution of inflammation (cytosol, secreted)	-1.78 (0.093733)	-2.82 (0.026954)	Promotes glioblastoma cell invasion (74). Involved in cancer-associated fibroblasts that promote tumorigenesis in lung cancer (75)

Table S8. Alterations in the expression of protein synthesis- and degradation-related human proteins in GBM xenografts treated for short and long periods with si-hVDAC1

LC-HR MS/MS analysis was performed as described in the Materials and Methods section. Differentially expressed proteins (p-value <0.05, FC ǀ1.5ǀ) were filtered and those proteins differentially expressed in tumors treated with si-NT- or si-hVDAC1 for short or long periods are presented. For each protein, the name, fold change (FC) and p-value, as well as its function, subcellular localization and relevance to cancer, are indicated.

**No.**	**Protein name** (UniProtKB)	**Proposed function (cell localization)**	**Short-term treatment si-hVDAC1/ si-NT FC (P value)**	**Long-term treatment si-hVDAC1/ si-NT FC (P value)**	**Relation to cancer**
1	**MID1 - E3 ubiquitin-protein ligase Midline-1** (O15344)	Possesses E3 ubiquitin ligase activity towards PP2A, calpain and the GLI3 regulator Fu, promoting their proteasomal degradation (cytoskeleton, cytosol)	-2.1 (0.441711)	-50.9 (0.013575)	Highly expressed in prostate cancer cells with aggressive phenotype (76)
2	**MRPL18 - Mitochondrial ribosomal protein L18** (Q9H0U6)	Mitochondrial ribosomal protein. Together with thiosulfate sulfur-transferase, acts as a mitochondrial import factor for cytosolic 5S rRNA (mitochondria)	-1.24 (0.4977670)	-2.63 (0.0386381)	Over-expressed in colon cancer (77)
3	**MRPL37 - Mitochondrial ribosomal protein L37** (Q9BZE1)	Mitochondrial ribosomal protein. Regulates mitochondrial protein translation (mitochondria)	-1.31 (0.491118)	-3.09 (0.045548)	Over-expressed in colon carcinomas (78)
4	**MRPL9 - 39S ribosomal protein L9, mitochondrial** (O43390)	Mitochondrial ribosomal protein having several functions, as poly(A) RNA binding, protein binding, structural constituent of ribosome (mitochondria)	-1.41 (0.661372)	-23.73 (0.0137302)	Up-regulated in CRC. Involved in cell growth, long-term colony formation and inhibition of apoptosis (79)
5	**NUB1 - NEDD8 ultimate buster 1** (Q9Y5A7)	Recruits FAT10- and NEDD8-conjugated proteins for proteasome-mediated degradation (nucleus)	-2.2 (0.389677)	-52.1 (0.01918)	Up-regulation of NUB1 inhibits proliferation of IFNα-resistant RCC cells (80)
6	**PSMA6 – Proteasome subunit alpha type-6** (P60900)	A structural component of the outer α rings of the 20S core proteasome (proteasome)	-6.84 (0.0261956)	-2.51 (0.295258)	Inhibits apoptosis and regulates cell cycle of lung cancer cells (81)
7	**RALY – RNA-binding protein Raly** (Q9UKM9)	Involved in mRNA metabolism (cytoplasm)	-1.40 (0.163783)	-2.23 (0.0215714)	Higher levels are associated with poor prognosis in ovarian, lung, bladder, brain and breast cancers, multiple myelomas and melanomas and in HCC (82,83)
8	**αNAC - Nascent polypeptide-associated complex subunit alpha** (E9PAV3)	A subunit of NAC, ribosome associated complex that interacts with the nascent peptide to protect it from proteolysis to facilitate its folding (cytosol)	-1.28 (0.534135)	-7.68 (0.0044573)	None reported

Table S9. Alterations in the expression of epigenetic regulation- and DNA structure-related human proteins in GBM xenografts treated for short and long periods with si-hVDAC1

LC-HR MS/MS analysis was performed as described in the Materials and Methods section. Differentially expressed proteins (p-value <0.05, FC |1.5|) were filtered and those proteins differentially expressed in tumors treated with si-NT- or si-hVDAC1 for short or long periods are presented. For each protein, the name, fold change (FC) and p-value, as well as its function, subcellular localization and relevance to cancer, are indicated.

No.	**Protein name** (UniProtKB)	**Proposed function (cell localization)**	**Short-term treatment si-hVDAC1/ si-NT FC (P value)**	**Long-term treatment si-hVDAC1/ si-NT FC (P value)**	**Relation to cancer**
1	**BZW1- Basic leucine zipper and W2 domain-containing protein 1** (Q7L1Q6)	Activates histone H4 gene transcription and serves as a co-regulator of other transcription factors for the control of cell cycling (cytosol, nucleus)	-1.53 (0.0912487)	-2.11 (0.0284313)	Facilitate growth of tumor cells in mucoepidermoid carcinoma (84)
2	**CBX5/HP1α - Chromobox protein homolog 5 / heterochromatin protein-1α** (P45973)	Component of heterochromatin that recognizes and binds histone H3 tails methylated at lysine 9 (H3K9me), leading to epigenetic repression (nucleus)	-1.76 (0.464606)	-9.24 (0.04653)	High expression was correlated to worsen relapse-free survival for all breast cancer patients(85)
3	**H1FX - Histone H1x** (Q92522)	Necessary for the condensation of nucleosome chains into higher-order structures (nucleus)	-1.48 (0.271339)	-3.57 (0.0189979)	Prognosticator in glioblastomas (86)
4	**HPF1- Histone PARylation factor 1** (Q9NWY4)	Promotes histone serine ADP-ribosylation in response to DNA damage (nucleus)	1.62 (0.558448)	-14.88 (0.0306001)	None reported
5	**MATR3 – Matrin 3** (P43243)	Involved in DNA damage response (nucleus)	-1.07 (0.835464)	-4.37 (0.0077896)	Mediates pro-survival effect in response to DNA damage in CRC (87)
6	**MAX – MYC associated factor X** (P61244)	Regulates gene transcription and cell proliferation (nucleus)	4.96 (0.0168612)	3.18 (0.0649824)	Involved in pathogenesis of neuroblastoma (88)
7	**CAP-D2 - Condensin complex subunit 1** (Q15021)	Regulatory subunit of the condensin I complex required for conversion of interphase chromatin into mitotic-like condensed chromosomes (nucleus)	-13.77 (0.037987)	-3.11 (0.384659)	None reported
8	**CAP-H - Condensin complex subunit 2** (Q15003)	Regulatory subunit (kleisin) of the condensin-1 complex that provides chromosomes with an additional level of organization (nucleus)	-1.13 (0.0616937)	-25.97 (0.038981)	None reported
9	**SMC2- Structural maintenance of chromosomes protein 2** (O95347)	A core subunit of the condensin complex required to condense the chromosomes at the onset of mitosis (nucleus)	-1.96 (0.339075)	-8.7 (0.0346239)	Depletion of SMC2 in CRC reduced proliferation (89)
10	**SMC4-Structural maintenance of chromosomes protein 4** (Q9NTJ3)	A core subunit of the condensin complex required to condense chromosomes at the onset of mitosis (nucleus)	-1.2 (0.511603)	-3.94 (0.00466)	Associated with tumor de-differentiation, advanced stage and vascular invasion of primary liver cancer (90)
11	**HP1BP3 – Heterochromatin protein 1 binding protein 3** (Q5SSJ5)	Involved in modulation of gene expression. Plays a vital and non-redundant role in viability and growth (nucleus)	-1.21 (0.438031)	-2.10 (0.0390518)	Marker of intrinsic 5-fluorouracil resistance in colorectal cancer (91)

Table S10. Alterations in the expression of epigenetic and DNA replication-related human proteins in GBM xenografts treated for short and long periods with si-hVDAC1

LC-HR MS/MS analysis was performed as described in the Materials and Methods section. Differentially expressed proteins (p-value <0.05, FC |1.5|) were filtered and those proteins differentially expressed in tumors treated with si-NT- or si-hVDAC1 for short or long periods are presented. For each protein, the name, fold change (FC) and p-value, as well as its function, subcellular localization and relevance to cancer, are indicated.

No.	**Protein name** (UniProtKB)	**Proposed function (cell localization)**	**Short-term treatment si-hVDAC1/ si-NT FC (P value)**	**Long-term treatment si-hVDAC1/ si-NT FC (P value)**	**Relation to cancer**
1	**FEN1 - Flap endonuclease 1** (P39748)	Regulates DNA replication, repair, recombination and transcription (mitochondria, nucleus)	-2.13 (0.058908)	-3.61 (0.018072)	Over-expressed in breast and gastric cancers and essential for proliferation. Involved in chemotherapy resistance (92)
2	**LARP7 – La ribonucleoprotein domain family, member-7** (Q4G0J3)	Regulates telomerase activity (nucleus)	-1.63 (0.725744)	-127.55 (0.0497922)	Functions as suppressor in breast cancer and gastric carcinoma (93,94)
3	**MCM3- DNA replication-licensing factor MCM3** (P25205)	Acts as component of the MCM2-7 complex, the putative replicative helicase (nucleus)	-2.11 (0.174867)	-5.4 (0.029871)	Proliferation marker of salivary gland tumors (95)
4	**MCM5- DNA replication licensing factor MCM5** (P33992)	Acts as component of the MCM2-7 complex, the putative replicative helicase (cytosol, nucleus)	-2.9 (0.141522)	-6.74 (0.048305)	Over-expressed in lung cancer metastasis (96)
5	**MCM7- DNA replication-licensing factor MCM7** (P33993)	Acts as component of the MCM2-7 complex which is the putative replicative helicase (nucleus)	-1.84 (0.239604)	-4.39 (0.041277)	Promotes tumor cell proliferation and tumorigenesis in HCC (97)
6	**RFC3- Replication factor C subunit 3** (P40938)	Elongation of primed DNA templates by DNA polymerase delta and epsilon requires the action of the accessory proteins PCNA and activator 1 (nucleus)	3.62 (0.131966)	-53.52 (0.009262)	Promotes metastasis in breast cancer. High expression levels are associated with poor prognosis (98)
7	**RFC4- Replication factor C subunit 4** (P35249)	Elongation of primed DNA templates by DNA polymerase delta and epsilon requires the action of PCNA and activator 1 (nucleus)	-1.89 (0.273506)	-4.97 (0.047627)	Increased expression levels in colorectal cancer (99)
8	**RRM2B- Ribonucleoside-diphosphate reductase subunit M2 B** (Q7LG56)	Plays a pivotal role in cell survival by repairing damaged DNA in a p53-dependent manner (nucleus)	3.17 (0.017337)	4.11 (0.017243)	Associated with aggressive clinicopathologic manifestations of breast cancer **(100)**
9	**SSBP1 - Single-stranded DNA-binding protein** (Q04837)	Binds preferentially and cooperatively to single strand-DNA. Involved in mitochondrial biogenesis (mitochondria)	-1.15 (0.629587)	-2.37 (0.041082)	Down-regulated in highly metastatic breast cancer cells. Inhibits tumor metastasis and EMT (101)
10	**SUB1 homolog / PC4 – Positive cofactor 4** (P53999)	Involved in DNA replication, DNA repair and transcription (nucleus)	-2.04 (0.098065)	-3.48 (0.0311694)	Up-regulated in metastatic prostate cancer [9] and astrocytoma (102)
11	**TOP1-DNA topoisomerase 1** (P11387)	Releases the supercoiling and torsional tension of DNA introduced during DNA replication and transcription (nucleus)	-1.56 (0.358155)	-4.15 (0.040935)	Involved in tumorigenesis (103)

References

1. Kelstrup, C. D., Young, C., Lavallee, R., Nielsen, M. L., and Olsen, J. V. (2012) Optimized fast and sensitive acquisition methods for shotgun proteomics on a quadrupole orbitrap mass spectrometer. *J Proteome Res* **11**, 3487-3497
2. Ueda, K., Saichi, N., Takami, S., Kang, D., Toyama, A., Daigo, Y., *et al.* (2011) A comprehensive peptidome profiling technology for the identification of early detection biomarkers for lung adenocarcinoma. *PLoS One* **6**, e18567
3. Guryca, V., Lamerz, J., Ducret, A., and Cutler, P. (2012) Qualitative improvement and quantitative assessment of N-terminomics. *Proteomics* **12**, 1207-1216
4. D'Arena, G., Laurenti, L., Capalbo, S., D'Arco, A. M., De Filippi, R., Marcacci, G., *et al.* (2006) Rituximab therapy for chronic lymphocytic leukemia-associated autoimmune hemolytic anemia. *Am J Hematol* **81**, 598-602
5. Max, D., Hesse, M., Volkmer, I., and Staege, M. S. (2009) High expression of the evolutionarily conserved alpha/beta hydrolase domain containing 6 (ABHD6) in Ewing tumors. *Cancer science* **100**, 2383-2389
6. Wang, X., Yang, K., Xie, Q., Wu, Q., Mack, S. C., Shi, Y., *et al.* (2017) Purine synthesis promotes maintenance of brain tumor initiating cells in glioma. *Nat Neurosci* **20**, 661
7. Wang, N.-n., Wang, L.-H., Li, Y., Fu, S.-Y., Xue, X., Jia, L.-N., *et al.* (2018) Targeting ALDH2 with disulfiram/copper reverses the resistance of cancer cells to microtubule inhibitors. *Exp Cell Res* **362**, 72-82
8. Lin, C.-C., Cheng, T.-L., Tsai, W.-H., Tsai, H.-J., Hu, K.-H., Chang, H.-C., *et al.* (2012) Loss of the respiratory enzyme citrate synthase directly links the Warburg effect to tumor malignancy. *Sci Rep* **2**, 785
9. Zhang, Y., Qian, Y., Zhang, J., Yan, W., Jung, Y.-S., Chen, M., *et al.* (2017) Ferredoxin reductase is critical for p53-dependent tumor suppression via iron regulatory protein 2. *Genes Dev* **31**, 1243-1256
10. Feng, Z., Li, J. N., Wang, L., Pu, Y. F., Wang, Y., and Guo, C. B. (2014) The prognostic value of glycerol-3-phosphate dehydrogenase 1-like expression in head and neck squamous cell carcinoma. *Histopathology* **64**, 348-355
11. Perryman, M. R., O'Neill, M. K., Keun, D. H., and Syed, D. N. (2017) PP61. THE ROLE OF NICOTINAMIDE METABOLISM IN CHEMOSENSITIVITY IN GLIOBLASTOMA MULTIFORME. *Neuro-Oncology* **19**, i17-i17
12. Shali, S., Yu, J., Zhang, X., Wang, X., Jin, Y., Su, M., *et al.* (2018) Ecto-5'-nucleotidase (CD73) is a potential target of hepatocellular carcinoma. *J Cell Physiol* **234**, 10248-10259
13. Bonnin, N., Armandy, E., Carras, J., Ferrandon, S., Battiston-Montagne, P., Aubry, M., *et al.* (2016) MiR-422a promotes loco-regional recurrence by targeting NT5E/CD73 in head and neck squamous cell carcinoma. *Oncotarget* **7**, 44023-44038
14. Pasdziernik, M., Kaltschmidt, B., Kaltschmidt, C., Klinger, C., and Kaufmann, M. (2009) On the cytotoxicity of HCR-NTPase in the neuroblastoma cell line SH-SY5Y. *BMC Res Notes* **2**, 102-102
15. Zhang, S., and Xie, C. (2017) The role of OXCT1 in the pathogenesis of cancer as a rate-limiting enzyme of ketone body metabolism. *Life Sci* **183**, 110-115
16. Phannasil, P., Thuwajit, C., Warnnissorn, M., Wallace, J. C., MacDonald, M. J., and Jitrapakdee, S. (2015) Pyruvate carboxylase is up-regulated in breast cancer and essential to support growth and invasion of MDA-MB-231 cells. *PLoS One* **10**, e0129848
17. Cai, F., Miao, Y., Liu, C., Wu, T., Shen, S., Su, X., *et al.* (2018) Pyrroline-5-carboxylate reductase 1 promotes proliferation and inhibits apoptosis in non-small cell lung cancer. *Oncology letters* **15**, 731-740
18. Schaefer, I.-M., Hornick, J. L., and Bovée, J. V. M. G. (2018) The role of metabolic enzymes in mesenchymal tumors and tumor syndromes: genetics, pathology, and molecular mechanisms. *Lab Invest* **98**, 414-426
19. Al-Khayal, K., ABDuLLA, M., Al-Obeed, O., Al Kattan, W., ZuBAIDI, A., Vaali-Mohammed, M.-A., *et al.* (2016) Identification of the TP53-induced glycolysis and apoptosis regulator in various stages of colorectal cancer patients. *Oncol Rep* **35**, 1281-1286
20. Saito, K., Uzawa, K., Endo, Y., Kato, Y., Nakashima, D., Ogawara, K., *et al.* (2006) Plasma membrane Ca2+ ATPase isoform 1 down-regulated in human oral cancer. *Oncol Rep* **15**, 49-55
21. Guo, F., Liu, Y., Li, Y., and Li, G. (2010) Inhibition of ADP-ribosylation factor-like 6 interacting protein 1 suppresses proliferation and reduces tumor cell invasion in CaSki human cervical cancer cells. *Mol Biol Rep* **37**, 3819-3825
22. Carvalho, K. C., Cunha, I. W., Rocha, R. M., Ayala, F. R., Cajaíba, M. M., Begnami, M. D., *et al.* (2011) GLUT1 expression in malignant tumors and its use as an immunodiagnostic marker. *Clinics (Sao Paulo, Brazil)* **66**, 965-972
23. Ancey, P.-B., Contat, C., and Meylan, E. (2018) Glucose transporters in cancer – from tumor cells to the tumor microenvironment. *FEBS* **285**, 2926-2943
24. Yang, Z., Ni, W., Cui, C., Qi, W., Piao, L., and Xuan, Y. (2018) Identification of LETM1 as a marker of cancer stem-like cells and predictor of poor prognosis in esophageal squamous cell carcinoma. *Hum Pathol* **81**, 148-156
25. Cazzaniga, A., Moscheni, C., Trapani, V., Wolf, F. I., Farruggia, G., Sargenti, A., *et al.* (2017) The different expression of TRPM7 and MagT1 impacts on the proliferation of colon carcinoma cells sensitive or resistant to doxorubicin. *Sci Rep* **7**, 40538
26. Franklin, R. B., Levy, B. A., Zou, J., Hanna, N., Desouki, M. M., Bagasra, O., *et al.* (2012) ZIP14 zinc transporter downregulation and zinc depletion in the development and progression of hepatocellular cancer. *J Gastrointest Cancer* **43**, 249-257
27. Varghese, S., Burness, M., Xu, H., Beresnev, T., Pingpank, J., and Alexander, H. R. (2007) Site-specific gene expression profiles and novel molecular prognostic factors in patients with lower gastrointestinal adenocarcinoma diffusely metastatic to liver or peritoneum. *Ann Surg Oncol* **14**, 3460-3471

28. Ohara, N., Haraguchi, N., Koseki, J., Nishizawa, Y., Kawai, K., Takahashi, H., *et al.* (2017) Low expression of the GOPC is a poor prognostic marker in colorectal cancer. *Oncology letters* **14**, 4483-4490
29. Kooi, I. E., Mol, B. M., Massink, M. P., de Jong, M. C., de Graaf, P., van der Valk, P., *et al.* (2016) A Meta-Analysis of Retinoblastoma Copy Numbers Refines the List of Possible Driver Genes Involved in Tumor Progression. *PLoS One* **11**, e0153323
30. Makise, M., Nakamura, H., and Kuniyasu, A. (2018) The role of vimentin in the tumor marker Nup88-dependent multinucleated phenotype. *BMC Cancer* **18**, 519
31. Li, J., Zhao, J., and Li, Y. (2017) Multiple biological processes may be associated with tumorigenesis under NUP88-overexpressed condition. *Genes, Chromosomes and Cancer* **56**, 117-127
32. Lee, J. H., Zhao, X. M., Yoon, I., Lee, J. Y., Kwon, N. H., Wang, Y. Y., *et al.* (2016) Integrative analysis of mutational and transcriptional profiles reveals driver mutations of metastatic breast cancers. *Cell Discov* **2**, 16025
33. Kumar, R. D., Searleman, A. C., Swamidass, S. J., Griffith, O. L., and Bose, R. (2015) Statistically identifying tumor suppressors and oncogenes from pan-cancer genome-sequencing data. *Bioinformatics (Oxford, England)* **31**, 3561-3568
34. van den Boom, J., Wolter, M., Blaschke, B., Knobbe, C. B., and Reifenberger, G. (2006) Identification of novel genes associated with astrocytoma progression using suppression subtractive hybridization and real-time reverse transcription-polymerase chain reaction. *Int J Cancer* **119**, 2330-2338
35. Andreeva, A. V., Kutuzov, M. A., and Voyno-Yasenetskaya, T. A. (2006) A ubiquitous membrane fusion protein αSNAP: a potential therapeutic target for cancer, diabetes and neurological disorders? *Expert Opinion on Therapeutic Targets* **10**, 723-733
36. Han, M., Xu, R., Wang, S., Yang, N., Ni, S., Zhang, Q., *et al.* (2018) Six-Transmembrane Epithelial Antigen of Prostate 3 Predicts Poor Prognosis and Promotes Glioblastoma Growth and Invasion. *Neoplasia* **20**, 543-554
37. Sotgia, F., Whitaker-Menezes, D., Martinez-Outschoorn, U. E., Salem, A. F., Tsirigos, A., Lamb, R., *et al.* (2012) Mitochondria "fuel" breast cancer metabolism: Fifteen markers of mitochondrial biogenesis label epithelial cancer cells, but are excluded from adjacent stromal cells. *Cell Cycle* **11**, 4390-4401
38. Liu, R.-T., Huang, C.-C., You, H.-L., Chou, F.-F., Hu, C.-C. A., Chao, F.-P., *et al.* (2002) Overexpression of tumor susceptibility gene TSG101 in human papillary thyroid carcinomas. *Oncogene* **21**, 4830
39. Oh, K. B., Stanton, M. J., West, W. W., Todd, G. L., and Wagner, K. U. (2007) Tsg101 is upregulated in a subset of invasive human breast cancers and its targeted overexpression in transgenic mice reveals weak oncogenic properties for mammary cancer initiation. *Oncogene* **26**, 5950
40. Heinrich, C., Keller, C., Boulay, A., Vecchi, M., Bianchi, M., Sack, R., *et al.* (2010) Copine-III interacts with ErbB2 and promotes tumor cell migration. *J Oncogene* **29**, 1598
41. Guo, P., Huang, J., Wang, L., Jia, D., Yang, J., Dillon, D. A., *et al.* (2014) ICAM-1 as a molecular target for triple negative breast cancer. *Proc Natl Acad Sci U S A.* **111**, 14710-14715
42. Huang, C., Li, N., Li, Z., Chang, A., Chen, Y., Zhao, T., *et al.* (2017) Tumour-derived Interleukin 35 promotes pancreatic ductal adenocarcinoma cell extravasation and metastasis by inducing ICAM1 expression. *Nature communications* **8**, 14035
43. Park, S., and James, C. D. (2003) Lanthionine Synthetase Components C-like 2 Increases Cellular Sensitivity to Adriamycin by Decreasing the Expression of P-Glycoprotein through a Transcription-mediated Mechanism. *Cancer Res* **63**, 723-727
44. Xu, K., Chen, G., Li, X., Wu, X., Chang, Z., Xu, J., *et al.* (2017) MFN2 suppresses cancer progression through inhibition of mTORC2/Akt signaling. *Scientific Reports* **7**, 41718
45. Cheng, J., Qian, D., Ding, X., Song, T., Cai, M., Dan, X., *et al.* (2018) High PGAM5 expression induces chemoresistance by enhancing Bcl-xL-mediated anti-apoptotic signaling and predicts poor prognosis in hepatocellular carcinoma patients. *Cell Death Dis* **9**, 991
46. Le, A. P., Huang, Y., Pingle, S. C., Kesari, S., Wang, H., Yong, R. L., *et al.* (2015) Plexin-B2 promotes invasive growth of malignant glioma. *Oncotarget* **6**, 7293-7304
47. Campos, T., Ziehe, J., Palma, M., Escobar, D., Tapia, J. C., Pincheira, R., *et al.* (2016) Rheb promotes cancer cell survival through p27Kip1-dependent activation of autophagy. *Mol Carcinog* **55**, 220-229
48. Gilkes, D. M., Xiang, L., Lee, S. J., Chaturvedi, P., Hubbi, M. E., Wirtz, D., *et al.* (2014) Hypoxia-inducible factors mediate coordinated RhoA-ROCK1 expression and signaling in breast cancer cells. *Proceedings of the National Academy of Sciences of the United States of America* **111**, E384-E393
49. Liu, J., Zhang, C., Wu, R., Lin, M., Liang, Y., Liu, J., *et al.* (2015) RRAD inhibits the Warburg effect through negative regulation of the NF-κB signaling. *Oncotarget* **6**, 14982-14992
50. Shang, R., Wang, J., Sun, W., Dai, B., Ruan, B., Zhang, Z., *et al.* (2016) RRAD inhibits aerobic glycolysis, invasion, and migration and is associated with poor prognosis in hepatocellular carcinoma. *Tumor Biology* **37**, 5097-5105
51. Chan, S. R., Vermi, W., Luo, J., Lucini, L., Rickert, C., Fowler, A. M., *et al.* (2012) STAT1-deficient mice spontaneously develop estrogen receptor α-positive luminal mammary carcinomas. *Breast Cancer Research* **14**, R16
52. Lesinski, G. B., Anghelina, M., Zimmerer, J., Bakalakos, T., Badgwell, B., Parihar, R., *et al.* (2003) The antitumor effects of IFN-α are abrogated in a STAT1-deficient mouse. *The Journal of clinical investigation* **112**, 170-180
53. Cao, H., Zhang, J., Liu, H., Wan, L., Zhang, H., Huang, Q., *et al.* (2016) IL-13/STAT6 signaling plays a critical role in the epithelial-mesenchymal transition of colorectal cancer cells. *Oncotarget* **7**, 61183-61198

54. Sheets, J., Iwanicki, M., Liu, J., Howitt, B., Hirsch, M., Gubbels, J., *et al.* (2016) SUSD2 expression in high-grade serous ovarian cancer correlates with increased patient survival and defective mesothelial clearance. *Oncogenesis* **5**, e264
55. Xu, Y., Miao, C., Jin, C., Qiu, C., Li, Y., Sun, X., *et al.* (2018) SUSD2 promotes cancer metastasis and confers cisplatin resistance in high grade serous ovarian cancer. *Experimental Cell Research* **363**, 160-170
56. Cheng, Y., Wang, X., Wang, P., Li, T., Hu, F., Liu, Q., *et al.* (2016) SUSD2 is frequently downregulated and functions as a tumor suppressor in RCC and lung cancer. *Tumour Biol* **37**, 9919-9930
57. Lin, J., He, Y., Chen, L., Chen, X., Zang, S., and Lin, W. (2018) MYLK promotes hepatocellular carcinoma progression through regulating cytoskeleton to enhance epithelial–mesenchymal transition. *Clin Exp Med* **18**, 523-533
58. Yang, Y., Yang, L., and Li, Y. (2018) Neuropilin-1 (NRP-1) upregulated by IL-6/STAT3 signaling contributes to invasion in pancreatic neuroendocrine neoplasms. *Human Pathology* **81**, 192-200
59. Zhang, G., Chen, L., Sun, K., Khan, A. A., Yan, J., Liu, H., *et al.* (2016) Neuropilin-1 (NRP-1)/GIPC1 pathway mediates glioma progression. *Tumour Biol* **37**, 13777-13788
60. Rafiei, S., Tiedemann, K., Tabariès, S., Siegel, P. M., and Komarova, S. V. (2015) Peroxiredoxin 4: A novel secreted mediator of cancer induced osteoclastogenesis. *Cancer Letters* **361**, 262-270
61. Zhu, H., Leiss, L., Yang, N., Rygh, C. B., Mitra, S. S., Cheshier, S. H., *et al.* (2017) Surgical debulking promotes recruitment of macrophages and triggers glioblastoma phagocytosis in combination with CD47 blocking immunotherapy. *Oncotarget* **8**, 12145-12157
62. Ruma, I. M. W., Kinoshita, R., Tomonobu, N., Inoue, Y., Kondo, E., Yamauchi, A., *et al.* (2018) Embigin Promotes Prostate Cancer Progression by S100A4-Dependent and-Independent Mechanisms. *Cancers (Basel)* **10**, 239
63. Jung, D. E., Kim, J. M., Kim, C., and Song, S. Y. (2016) Embigin is overexpressed in pancreatic ductal adenocarcinoma and regulates cell motility through epithelial to mesenchymal transition via the TGF-beta pathway. *Mol Carcinog* **55**, 633-645
64. Konstantinopoulos, P. A., Karamouzis, M. V., Papatsoris, A. G., and Papavassiliou, A. G. (2008) Matrix metalloproteinase inhibitors as anticancer agents. *The International Journal of Biochemistry & Cell Biology* **40**, 1156-1168
65. Antony-Debré, I., Bluteau, D., Itzykson, R., Baccini, V., Renneville, A., Boehlen, F., *et al.* (2012) MYH10 protein expression in platelets as a biomarker of RUNX1 and FLI1 alterations. *Blood* **120**, 2719-2722
66. Cai, X., Ding, H., Liu, Y., Pan, G., Li, Q., Yang, Z., *et al.* (2017) Expression of HMGB2 indicates worse survival of patients and is required for the maintenance of Warburg effect in pancreatic cancer. *Acta biochimica et biophysica Sinica* **49**, 119-127
67. Morath, I., Hartmann, T. N., and Orian-Rousseau, V. (2016) CD44: More than a mere stem cell marker. *The International Journal of Biochemistry & Cell Biology* **81**, 166-173
68. Senbanjo, L. T., and Chellaiah, M. A. (2017) CD44: A Multifunctional Cell Surface Adhesion Receptor Is a Regulator of Progression and Metastasis of Cancer Cells. *Frontiers in cell and developmental biology* **5**, 18-18
69. Awad, W., Logan, D. T., and Mani, K. (2014) GPC1 (glypican 1). *Atlas Genet Cytogenet Oncol Haematol* **18**, 461-464
70. Li, J., Chen, Y., Guo, X., Zhou, L., Jia, Z., Peng, Z., *et al.* (2017) GPC 1 exosome and its regulatory mi RNA s are specific markers for the detection and target therapy of colorectal cancer. *J Cell Mol Med* **21**, 838-847
71. Koshizuka, K., Hanazawa, T., Kikkawa, N., Arai, T., Okato, A., Kurozumi, A., *et al.* (2017) Regulation of ITGA3 by the anti-tumor miR-199 family inhibits cancer cell migration and invasion in head and neck cancer. *Cancer Sci* **108**, 1681-1692
72. Feng, L., Ma, J., Ji, H., Liu, Y., and Hu, W. (2017) miR-330-5p suppresses glioblastoma cell proliferation and invasiveness through targeting ITGA5. *Bioscience reports* **37**, BSR20170019
73. Zheng, W., Jiang, C., and Li, R. (2016) Integrin and gene network analysis reveals that ITGA5 and ITGB1 are prognostic in non-small-cell lung cancer. *OncoTargets and therapy* **9**, 2317-2327
74. Toussaint, L. G., 3rd, Nilson, A. E., Goble, J. M., Ballman, K. V., James, C. D., Lefranc, F., *et al.* (2012) Galectin-1, a gene preferentially expressed at the tumor margin, promotes glioblastoma cell invasion. *Mol Cancer* **11**, 32
75. Hsu, Y. L., Hung, J. Y., Chiang, S. Y., Jian, S. F., Wu, C. Y., Lin, Y. S., *et al.* (2016) Lung cancer-derived galectin-1 contributes to cancer associated fibroblast-mediated cancer progression and immune suppression through TDO2/kynurenine axis. *Oncotarget* **7**, 27584-27598
76. Winter, J., Basilicata, M. F., Stemmler, M. P., and Krauss, S. (2016) The MID1 protein is a central player during development and in disease. *Frontiers in bioscience (Landmark edition)* **21**, 664-682
77. Kumar, K. U., Srivastava, S. P., and Kaufman, R. J. (1999) Double-Stranded RNA-Activated Protein Kinase (PKR) Is Negatively Regulated by 60S Ribosomal Subunit Protein L18. *Mol Cell Biol* **19**, 1116-1125
78. Loging, W. T., and Reisman, D. (1999) Elevated expression of ribosomal protein genes L37, RPP-1, and S2 in the presence of mutant p53. *Cancer epidemiology, biomarkers & prevention : a publication of the American Association for Cancer Research, cosponsored by the American Society of Preventive Oncology* **8**, 1011-1016
79. Baik, I. H., Jo, G. H., Seo, D., Ko, M. J., Cho, C. H., Lee, M. G., *et al.* (2016) Knockdown of RPL9 expression inhibits colorectal carcinoma growth via the inactivation of Id-1/NF-kappaB signaling axis. *International journal of oncology* **49**, 1953-1962
80. Tan, K.-L., and Pezzella, F. (2016) Inhibition of NEDD8 and FAT10 ligase activities through the degrading enzyme NEDD8 ultimate buster 1: A potential anticancer approach. *Oncology letters* **12**, 4287-4296

81. Kakumu, T., Sato, M., Goto, D., Kato, T., Yogo, N., Hase, T., *et al.* (2017) Identification of proteasomal catalytic subunit PSMA 6 as a therapeutic target for lung cancer. *Cancer Sci* **108**, 732-743
82. Tsofack, S. P., Garand, C., Sereduk, C., Chow, D., Aziz, M., Guay, D., *et al.* (2011) NONO and RALY proteins are required for YB-1 oxaliplatin induced resistance in colon adenocarcinoma cell lines. *Molecular cancer* **10**, 145
83. Zhu, Z., Zhang, Y., Huang, C., Tang, Y., Sun, C., Ju, W., *et al.* (2018) Overexpression of RALY promotes migration and predicts poor prognosis in hepatocellular carcinoma. *Cancer Manag Res* **10**, 5559-5572
84. Li, S., Chai, Z., Li, Y., Liu, D., Bai, Z., Li, Y., *et al.* (2009) BZW1, a novel proliferation regulator that promotes growth of salivary muocepodermoid carcinoma. *Cancer Letters* **284**, 86-94
85. Liang, Y.-K., Lin, H.-Y., Chen, C.-F., and Zeng, D. (2017) Prognostic values of distinct CBX family members in breast cancer. *Oncotarget* **8**, 92375-92387
86. Sepsa, A., Levidou, G., Gargalionis, A., Adamopoulos, C., Spyropoulou, A., Dalagiorgou, G., *et al.* (2015) Emerging role of linker histone variant H1x as a biomarker with prognostic value in astrocytic gliomas. A multivariate analysis including trimethylation of H3K9 and H4K20. *PloS one* **10**, e0115101-e0115101
87. Chaudhary, R., Gryder, B., Woods, W. S., Subramanian, M., Jones, M. F., Li, X. L., *et al.* (2017) Prosurvival long noncoding RNA PINCR regulates a subset of p53 targets in human colorectal cancer cells by binding to Matrin 3. *Elife* **6**, e23244
88. Wenzel, A., and Schwab, M. J. E. J. o. C. (1995) The mycN/max protein complex in neuroblastoma. Short review. *Eur J Cancer* **31**, 516-519
89. Dávalos, V., Súarez-López, L., Castaño, J., Messent, A., Abasolo, I., Fernandez, Y., *et al.* (2012) Human SMC2 protein, a core subunit of human condensin complex, is a novel transcriptional target of the WNT signaling pathway and a new therapeutic target. *J Biol Chem* **287**, 43472-43481
90. Zhou, B., Yuan, T., Liu, M., Liu, H., Xie, J., Shen, Y., *et al.* (2012) Overexpression of the structural maintenance of chromosome 4 protein is associated with tumor de-differentiation, advanced stage and vascular invasion of primary liver cancer. *Oncol Rep* **28**, 1263-1268
91. Hadac, J. N., Miller, D. D., Grimes, I. C., Clipson, L., Newton, M. A., Schelman, W. R., *et al.* (2016) Heterochromatin Protein 1 Binding Protein 3 Expression as a Candidate Marker of Intrinsic 5-Fluorouracil Resistance. *Anticancer research* **36**, 845-852
92. He, L., Zhang, Y., Sun, H., Jiang, F., Yang, H., Wu, H., *et al.* (2016) Targeting DNA Flap Endonuclease 1 to Impede Breast Cancer Progression. *EBioMedicine* **14**, 32-43
93. Ji, X., Lu, H., Zhou, Q., and Luo, K. (2014) LARP7 suppresses P-TEFb activity to inhibit breast cancer progression and metastasis. *eLife* **3**, e02907-e02907
94. Cheng, Y., Jin, Z., Agarwal, R., Ma, K., Yang, J., Ibrahim, S., *et al.* (2012) LARP7 is a potential tumor suppressor gene in gastric cancer. *Laboratory Investigation* **92**, 1013
95. Ashkavandi, Z. J., Najvani, A. D., Tadbir, A. A., Pardis, S., Ranjbar, M. A., and Ashraf, M. J. (2013) MCM3 as a novel diagnostic marker in benign and malignant salivary gland tumors. *Asian Pac J Cancer Prev* **14**, 3479-3482
96. Liu, Y.-Z., Wang, B.-S., Jiang, Y.-Y., Cao, J., Hao, J.-J., Zhang, Y., *et al.* (2017) MCMs expression in lung cancer: implication of prognostic significance. *J Cancer* **8**, 3641-3647
97. Qu, K., Wang, Z., Fan, H., Li, J., Liu, J., Li, P., *et al.* (2017) MCM7 promotes cancer progression through cyclin D1-dependent signaling and serves as a prognostic marker for patients with hepatocellular carcinoma. *Cell Death Dis* **8**, e2603
98. He, Z.-Y., Wu, S.-G., Peng, F., Zhang, Q., Luo, Y., Chen, M., *et al.* (2017) Up-regulation of RFC3 promotes triple negative breast cancer metastasis and is associated with poor prognosis Via EMT. *Transl Oncol* **10**, 1-9
99. Xiang, J., Fang, L., Luo, Y., Yang, Z., Liao, Y., Cui, J., *et al.* (2014) Levels of human replication factor C4, a clamp loader, correlate with tumor progression and predict the prognosis for colorectal cancer. *J Transl Med* **12**, 320
100. Xue, L., Liu, X., Wang, Q., Liu, C. Q., Chen, Y., Jia, W., *et al.* (2018) Ribonucleotide reductase subunit M2B deficiency leads to mitochondrial permeability transition pore opening and is associated with aggressive clinicopathologic manifestations of breast cancer. *American journal of translational research* **10**, 3635-3649
101. Jiang, H.-L., Sun, H.-F., Gao, S.-P., Li, L.-D., Huang, S., Hu, X., *et al.* (2016) SSBP1 suppresses TGFβ-driven epithelial-to-mesenchymal transition and metastasis in triple-negative breast cancer by regulating mitochondrial retrograde signaling. *Cancer Res* **76**, 952-964
102. Chen, L., Du, C., Wang, L., Yang, C., Zhang, J.-r., Li, N., *et al.* (2014) Human positive coactivator 4 (PC4) is involved in the progression and prognosis of astrocytoma. *Journal of the Neurological Sciences* **346**, 293-298
103. K Kathiravan, M., N Kale, A., and Nilewar, S. (2016) Discovery and development of topoisomerase inhibitors as anticancer agents. *Mini Rev Med Chem* **16**, 1219-1229
